# Deciphering the role of glycine betaine in enhancing plant performance and defense mechanisms against environmental stresses

**DOI:** 10.3389/fpls.2025.1582332

**Published:** 2025-07-23

**Authors:** Farwa Basit, Mohammed Alyafei, Faisal Hayat, Wasef Al-Zayadneh, Ali El-Keblawy, Saad Sulieman, Mohamed S. Sheteiwy

**Affiliations:** ^1^ Department of Biology, College of Science and Technology, Wenzhou-Kean University, Wenzhou, China; ^2^ Department of Integrative Agriculture, College of Agriculture and Veterinary Medicine, United Arab Emirates University, Al Ain, United Arab Emirates; ^3^ Department of Applied Biology, College of Sciences, University of Sharjah, Sharjah, United Arab Emirates; ^4^ Department of Agronomy, Faculty of Agriculture, University of Khartoum, Khartoum North, Sudan

**Keywords:** osmolytes, stress tolerance, biosynthesis, reactive oxygen species (ROS), stress signaling network

## Abstract

In the context of climate change, abiotic stresses are recognized as significant environmental challenges that limit agricultural productivity globally. These conditions disrupt normal plant growth and development processes. The ability of plants to tolerate these stressors is linked to their resilience mechanisms. Glycine betaine (GB), also known as betaine, is a derivative of methylated glycine identified in numerous plant species as a substance that mitigates the detrimental effects of stressful environments. GB is synthesized in the cytosol as an initial response to abiotic stress, and signaling molecules, such as jasmonic acid and methyl jasmonate, primarily initiate its production. Recent studies have highlighted their role in stimulating GB synthesis and its subsequent accumulation. The concentration of GB within a plant system can effectively indicate tolerance levels, ultimately contributing to the understanding of resilience mechanisms. GB plays a crucial role in reducing the accumulation and detoxification of reactive oxygen species (ROS), which aids in restoring photosynthesis and alleviating oxidative stress. It contributes to the stabilization of membranes and macromolecules and is essential for the protection and stabilization of photosynthetic components, such as ribulose-1,5-bisphosphate carboxylase/oxygenase, photosystem II, and quaternary enzyme and protein complex structures, under environmental stress conditions. Furthermore, GB can enhance stress tolerance even at minimal concentrations by activating the genes associated with stress defense mechanisms. Recent studies have demonstrated that the application of GB can protect against environmental challenges, thereby improving both crop yield and quality. This review concentrates on the role of GB in promoting abiotic stress tolerance and explores potential strategies for engineering GB biosynthesis in plants.

## 1 Introduction

Extreme weather is one of the main concerns in the twenty-first century. According to several available data, the Earth’s average surface temperature has risen by roughly 1.18°C since the late 19th century and is expected to rise by an additional 0.2°C every decade for the next 30 years (Bailey-Serres et al., 2019). The beginning and end of the crop growing season have changed significantly over the past 20 years, which has decreased crop output, reduced the amount of fresh water available for irrigation, and exacerbated biodiversity loss (Brown and Cunningham, 2019). This, in turn, threatens available natural resources, in addition to having a negative impact on crop production. Severe weather patterns and various abiotic stressors, including salt, cold, heat, freezing, and draft flooding, have decreased crop yield and food security worldwide. Urbanization, global population growth, and land use patterns have rapidly changed as a result of industrialization. Providing food to the growing population, even if the amount of land under cultivation is decreasing, is one of the most significant obstacles in the scientific community. Thus, it is essential to create climate-resilient crops, and the only way to ensure a continuous supply of food worldwide is to develop environmentally friendly techniques for managing plant stress (Basit et al., 2024b; Naeem et al., 2024).

The production and accumulation of suitable solutes are among the most well-established stress-responsive processes in plants. Small organic metabolites that are water soluble and membrane-impermeable are compatible solutes. They can accumulate significantly in the cytoplasm at high concentrations (0.2 M), corresponding to stress responses (Kurepin et al., 2015). Numerous prokaryotic and eukaryotic cells, from bacteria to higher species such as plants and animals, have been shown to contain compatible osmolytes, and these cells exhibit a great deal of chemical variety among different organisms (Rasheed et al., 2024). Numerous compatible solutes have been found in plant cellular systems, such as quaternary amines (betaine, polyamines, and dimethyl sulfoniopropionate), sugars (trehalose), polyols, including sugar alcohols (mannitol, sorbitol, pinitol, glycerol, and galactinol), amino acids (proline, glutamate, glutamine, and alanine), and their derivatives (ectoine and hydroxyectoine) (Khan et al., 2009; Narwal et al., 2024).

Glycine betaine (GB), a low-molecular-weight molecule with high solubility and low viscosity, can accumulate significantly in the photosynthetic apparatus, including chloroplasts and plastids (Ali et al., 2020). Because of these exceptional qualities, GB is among the most effective osmoprotectants (Annunziata et al., 2019). According to previous reports, the degree of stress tolerance is generally related to the buildup of the GB (**Table 1**). However, several plants do not accumulate GB under stressful or normal conditions (Zulfiqar et al., 2022). According to previous reports, GB is one of the most suitable solutes for effectively protecting plants from a variety of abiotic stressors (Ali et al., 2020; Zulfiqar et al., 2022). Plants’ ability to withstand abiotic stress can be enhanced using exogenous GB application and transgenic plant techniques. GB is easily absorbed by the root or leaf tissue when applied exogenously through nutrient or foliar spray (Ibrahim et al., 2023). It also effectively stabilizes the quaternary structure of proteins and enzymes, oxygen-evolving photosystem II (PSII), and components of the photosynthetic machinery such as ribulose-1,5-biphosphate carboxylase/oxygenase (RuBisCO). GB helps to stabilize the cellular structures, particularly membranes and proteins, under conditions of high salinity and temperature stress. By maintaining membrane integrity and enzyme function, GB enhances plant resilience to abiotic stresses (Tian et al., 2017; Wang et al., 2019).

**Table 1:** The positive impact of glycine betaine (GB) supplementation on various plant species under different abiotic stresses.

Plant Species	Stress	GB's role in plants	Reference
Maize	Salt	Foliar sprays of Cu and Zn mitigated salt stress by promoting GB, Pro, amino acid, and sugar accumulation	(Iqbal et al., 2018)
Tomato	Salt	Symbiosis with *Piriformospora indica* promoted the accumulation of glycine betaine.	(Ghorbani et al., 2018)
Wheat	Drought	Parental drought priming enhanced glycine betaine accumulation and improved drought tolerance in offspring seedlings.	(Wang et al., 2018)
Oleander	Salt/Drought	The accumulation of glycine betaine activated the antioxidant machinery and restricted the transport of toxic ions from roots to shoots.	(Kumar et al., 2017)
Black goji berry	Salt	The induction of *AMADH1* during salt stress stimulated glycine betaine accumulation.	(Liu et al., 2018)
Wheat	Salt/Drought	Osmopriming with CaCl_2_ enhanced glycine betaine accumulation, trans-generational drought memory, and salt tolerance.	(Tabassum et al., 2017)
Cotton	Cd	Enhanced transpiration rate, water efficiency, and reduced malondialdehyde (MDA) content, electrolyte leakage, and Lowered cadmium (Cd) accumulation in leaves, stems, and roots.	(Farooq et al., 2016; Farooq et al., 2024)
Sugarcane	Chilling	Improved potassium (K^+^) and calcium (Ca²^+^) nutrition, increased free proline (Pro) and glycine betaine (GB) content. Also, elevated levels of soluble sugars.	(Rasheed et al., 2010)
Tomato	Waterlogging	Decreased MDA content, membrane injury, and Na contents by increasing K and Ca accumulation	(Rasheed et al., 2018)
Sorghum	Chromium	Reduce the oxidative stress	(Kumar, 2021)
Chickpea	Chromium	GB improved morpho-physiological and biochemical responses	(Singh et al., 2022)
Mung bean	Nickle	Improved physiological attributes	(Shahzad et al., 2023)
Cowpea	Cadmium	Reduced Cd uptake and improved plant physiological attributes	(Sadeghipour, 2020)
Rice	Aluminum	Improved plant growth, biomass, and photosynthesis	(Zhang et al., 2021)
Brassica	Chromium	GB improved morpho-physiological attributes and photosynthetic machinery	(Ahmad et al., 2020)
Mung bean	Chromium	Reduced Cr uptake and enhanced antioxidant activities	(Jabeen et al., 2016)
Soybean	Heat and salinity	Reduced phytotoxic impact and regulated redox homeostasis, physiological and molecular responses	(Bilal et al., 2023)
Beet	Cadmium	Enhanced antioxidant defense mechanism of plants	(Badawy et al., 2024)

Abiotic stress often triggers the overproduction of reactive oxygen species (ROS), which can result in oxidative stress (Basit et al., 2022a, b; Rao et al., 2025). GB has been found to have a positive effect in reducing oxidative damage caused by abiotic stressors in plants. According to Alcázar et al. (2010), exogenous hydroxyl radicals (OH^•^) caused a significant, dose-dependent outflow of K^+^ ions from epidermal cells in the elongation zone when applied to Arabidopsis roots. Nevertheless, this outflow of K^+^ ions was considerably decreased when GB was added to the incubation medium at a concentration of 5 mM. Exogenously administered GB also decreased cell membrane damage in cotton (Cheng et al., 2018) and the chilling-induced generation of H_2_O_2_ (Su and Wu, 2004) in tomato plants. By activating or stabilizing ROS-scavenging and/or suppressing the generation of ROS through other mechanisms, GB lessens the harmful consequences of oxidative stress even if it does not directly scavenge ROS. Fewer studies, nevertheless, have noted how ROS regulates plant growth and development and confers stress tolerance through its signaling function at lower cellular concentrations (Ali et al., 2020). The antioxidant system, which consists of both enzymatic and non-enzymatic components that scavenge or detoxify ROS (Ali et al., 2020; Zulfiqar et al., 2022), is one of the remarkable mechanisms that plants have evolved to maintain the cellular redox state and return oxidized macromolecules to their previous reduced states (Tian et al., 2017).

Thus, in this review, we have highlighted the biosynthesis, regulation, and protective mechanisms of GB in plants, emphasizing its various mechanisms for improving abiotic stress tolerance in plants. Additionally, we have provided information on how it can boost the growth and yield of different crop plants under stressful circumstances, as well as its synergistic effects with other genes that defend against stress. Furthermore, we discussed the interactive role of GB with phytohormones and other signaling networks to confer plant resistance to abiotic stress in order to boost productivity.

## 2 Biosynthesis and regulation mechanism of glycine betaine

### 2.1 Biosynthesis mechanism of glycine betaine in plants

Numerous data on GB synthesis in plants have been gathered, indicating that the cytoplasm, peroxisomes, and chloroplasts are the locations of GB production; nevertheless, the pathways by which GB is degraded remain unclear (Valenzuela-Soto and Figueroa-Soto, 2019). GB synthesis has been observed to influence the production of crucial plant growth regulators (PGRs), including auxin and ethylene (Annunziata et al., 2019). In the process of elucidating the synthesis of GB, it was discovered that phospho-ethanolamine N-methyltransferase (PEAMT), a cytosolic enzyme, catalyzes three consecutive adenosyl-methionine-dependent methylations of phospho-ethanolamine, which leads to the production of phospho-choline. Choline is subsequently produced from PEAMT-induced phosphocholine (PC), which has been proposed as the initial step in GB production (Ali et al., 2020). Plant-specific processes and distinct mechanisms are used to produce choline from PC. PC is promptly dephosphorylated to choline in spinach, but it is first incorporated into phosphatidylcholine in tobacco and then transformed into choline (McNeil et al., 2001). The bulk of the metabolic flux to choline occurs through PEAMT, which is subject to feedback regulation by PC. Therefore, PEAMT is a prime candidate for the genetic modification of plants to improve choline production (Annunziata et al., 2019).

**Figure 1:**
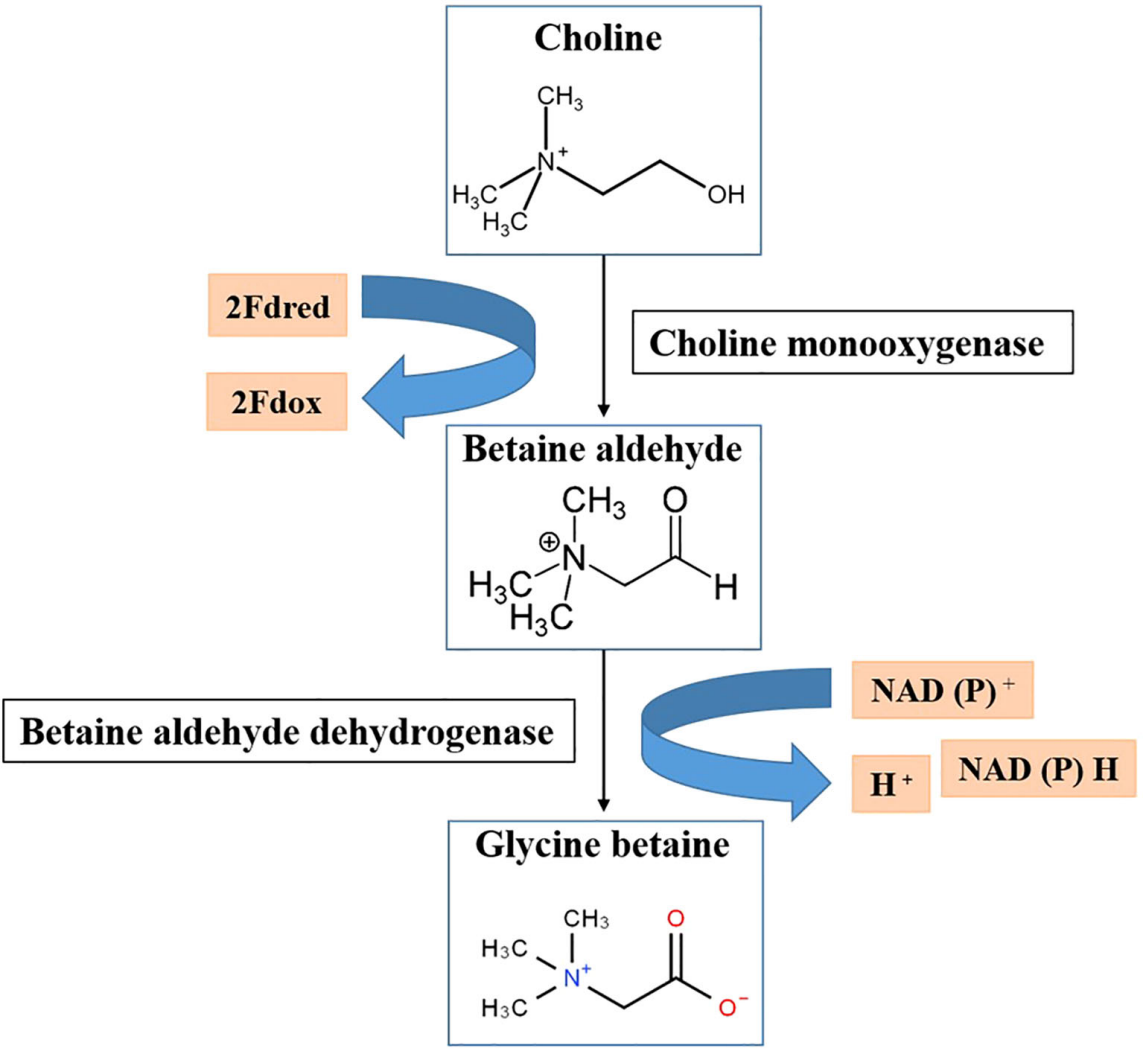
Biosynthesis of glycine betaine (GB) in plants. GB is synthesized from choline in two steps: choline is first converted to betaine aldehyde by choline monooxygenase (CMO), and then betaine aldehyde is further converted to GB by betaine aldehyde dehydrogenase (BADH). This pathway is crucial for stress tolerance, as GB stabilizes cellular structures under abiotic stress conditions. 2Fdred, 2Fe-ferredoxin reductase; 2Fdox, 2Fe-ferredoxin oxidized.

Choline is converted into GB by a process known as the GB biosynthesis pathway, which can be one-step in certain organisms, such as *Arthrobacter Spp*., or two-step in most organisms including plants. Members of the two angiosperm families Chenopodiaceae and Gramineae provided the first information regarding this route in higher plants. In these plants, choline is converted to GB in two steps with an intermediate called betaine aldehyde (Valenzuela-Soto and Figueroa-Soto, 2019). The oxidation of choline to betaine aldehyde in plants is mediated by choline monooxygenase (CMO) in the first stage, whereas the subsequent oxidation of betaine aldehyde is mediated by betaine aldehyde dehydrogenase in the second step (**Figure 1**). According to Weigel et al. (1986), the two enzymes involved in this process are BADH (an NAD^+^-dependent betaine aldehyde dehydrogenase) and CMO (a ferredoxin-dependent cholinexygenase). In certain plants, the chloroplast stroma contains both enzymes (Annunziata et al., 2019). There is evidence of a peroxisomal NADPH-dependent CMO in certain other plants, such as barley, whereas betaine aldehyde dehydrogenase (BADH) is found in both the cytosol and chloroplasts (Annunziata et al., 2019; Valenzuela-Soto and Figueroa-Soto, 2019).

Despite having orthologs of both *CMO* and *BADH*, rice is regarded as a typical plant species that does not accumulate GB. Northern blotting showed increased *OsCMO* expression of the rice gene *OsCMO*. Higher GB content and improved resistance to salt stress are the outcomes of heterologous transgenic expression of *OsCMO* in tobacco. Although it was infrequently accumulated in wild-type plants, immunoblotting showed that a functional *OsCMO* protein of the proper size was present in transgenic tobacco. When rice seedlings were exposed to salt stress, a significant number of abridged *OsCMO* proteins were produced, indicating a deficiency in functional *OsCMO* (Sharma et al., 2023).

### 2.2 Regulation mechanism of glycine betaine biosynthesis in plants

According to previous reports, several plant-catalyzing enzymes use betaine aldehyde to synthesize GB from choline. According to previous reports, CMO, a ferredoxin-dependent stromal enzyme, is the initial stage of GB synthesis in spinach (Wu et al., 2023). This enzyme, also known as an unusual iron-sulfur (Fe-S) enzyme, has been shown to be triggered in spinach under salinity stress. The osmoregulatory GB production pathway involves the GB aldehyde dehydrogenase gene (gbsA), based on an investigation (**Table 2**). The GbsA enzyme is selective for GB aldehyde and prefers NAD^+^ as compared to NADP^+^ as a cofactor (Ghorbani et al., 2023). It was shown that the GbsA enzyme’s activity increased GB levels, which in turn conferred high salt tolerance. Based on emerging research, gbsA is now known as the betaine aldehyde dehydrogenase gene (BADH). Additionally, GB-induced abiotic stress tolerance in watermelon suspension cells is mediated by PGR-mediated signaling. The conserved CGTCA pattern found in the promoter regions of the *CMO* and *BADH* genes of watermelon, which are involved in GB production, responds to methyl jasmonate (MeJA) (Sharma et al., 2023). Additionally, MeJA was successful in eliciting the expression of both *CMO* and *BADH*, which resulted in the buildup of GB, comparable to the osmotic stress induced by mannitol. Furthermore, GB buildup is considerably reduced when ibuprofen, an inhibitor of JA production, is administered prior to osmotic stress (Xu et al., 2018).

**Table 2:** Genetic engineering approaches to enhance glycine betaine (GB) synthesis in various plant species.

Plant Species	Gene (s) modified	Gene source	Method of Modification	Outcome	Reference
*Nicotiana tabacum*	*PEAMT* (phosphoethanolamine N-methyltransferase)	*Arabidopsis thaliana*	Nuclear transformation	Increased choline and GB levels; enhanced drought and salinity tolerance	(McNeil et al., 2001)
*Arabidopsis thaliana*	*codA* (choline oxidase)	*Arthrobacter globiformis*	Agrobacterium-mediated transformation	Increased GB accumulation and improved salt tolerance	(Sakamoto and Murata, 2002)
*Solanum tuberosum*	*codA* (choline oxidase)	*Arthrobacter globiformis*	Plastid transformation	Enhanced GB accumulation in chloroplasts; improved low-temperature resistance and photosynthetic performance	(Zhang et al., 2020)
*Nicotiana tabacum*	*betA* (choline dehydrogenase)	*Escherichia coli*	Agrobacterium-mediated transformation	Enhanced drought and salinity tolerance	(Holmström et al., 2000)
*Oryza sativa*	*codA*	*A. globiformis*	Agrobacterium-mediated transformation	Accumulated GB in chloroplasts and improved salt tolerance	(Mohanty et al., 2002)
*Zea mays* (maize)	*codA* (choline oxidase)	*Arthrobacter globiformis*	Nuclear transformation	Improved salt tolerance through enhanced GB synthesis and Na⁺ homeostasis	(Zhu et al., 2022)
*Brassica juncea*	*codA*	*A. globiformis*	Agrobacterium-mediated transformation	Improved drought resistance and photosynthetic efficiency	(Prasad et al., 2000)
*Glycine max*	*CMO* (choline monooxygenase), *BADH*	Spinach & Sugar beet	Agrobacterium-mediated transformation	Synergistic effect on GB synthesis and stress tolerance	(Sulpice et al., 2009)
*Zea mays*	*codA*	*A. globiformis*	Particle bombardment	Increased GB levels and better tolerance to oxidative stress	(Quan et al., 2004)
*Glycyrrhiza uralensis*	–	–	Exogenous GB application	Enhanced salt tolerance through modulation of multiple pathways	(Liang et al., 2024)
*Populus spp.*	*codA*	*A. globiformis*	Agrobacterium-mediated transformation	Improved water-use efficiency and salinity resistance	(Kathuria et al., 2009)
*Triticum aestivum*	*codA*	*A. globiformis*	Biolistic transformation	GB accumulation and enhanced growth under salt stress	(Zhang et al., 2020)
*Oryza sativa* (rice)	*codA* (choline oxidase)	*Arthrobacter globiformis*	Nuclear transformation	Enhanced GB accumulation; improved drought and salinity tolerance	(Hafez et al., 2021)

Gao et al. (2020) demonstrated that changes in lipid metabolism and GB synthesis play a key role in the development of salt tolerance in the halophytic seashore paspalum grass (*Paspalum vaginatum*) by regulating choline and genotypic variants. Through increases in digalactosyl diacylglycerol levels and phosphatidylinositol and phosphatidic acid synthesis, upregulation of GB synthesis led to choline-induced salt tolerance in the grass, which in turn led to changes in lipid metabolism.

## 3 Protective mechanism of glycine betaine in plants

### 3.1 Protective role of glycine betaine for translational and transcriptional machinery

Reinforcement of photosynthesis, osmoregulation, membrane integrity, detoxification of reactive oxygen radicals produced under stress conditions, and maintenance of the native structure of proteins and enzymes are suggested mechanisms for GB-mediated abiotic stress resistance (Hossain et al., 2021). However, due of the modest levels of GB accumulation in transgenic plants, these pathways might not fully account for the documented stress tolerance in these plants. Similar to plant hormones, the effective concentrations of GB upon absorption of exogenously applied GB or as a result of genetically modified GB production *in vivo* are within the millimolar range (Figueroa-Soto and Valenzuela-Soto, 2018). Therefore, it is equally logical to hypothesize that GB’s role in protecting the transcriptional and translational machinery may be mediated by activation of the expression of particular genes under a stress environment, whose products contribute to the establishment of stress tolerance (Hossain et al., 2021). GB might act as a modulator or signaling molecule under environmental stress, increasing the expression of genes related to osmoprotection, antioxidant defense, and protein stability. These genes produce antioxidant enzymes, heat shock proteins, molecular chaperones, and other defense mechanisms that preserve the integrity of ribosomes, RNA polymerases, and related regulatory proteins. Moreover, GB helps to preserve the structure and function of macromolecular complexes i.e., transcription factors and ribosomes. By protecting these complexes from denaturation or aggregation during abiotic stress, GB ensures the continuity of gene expression and protein synthesis, thereby enhancing cellular stress tolerance. This dual functionality positions GB as a key osmoprotectant and regulatory molecule in stress adaptation mechanisms (Sakamoto and Murata, 2002).

These gene products enhance the organism’s general ability to withstand stress by reducing oxidative damage and stabilizing macromolecular structures. Furthermore, GB might have an impact on transcription factors and epigenetic regulators, which would further optimize the cellular reaction to stress. As a result, its function goes beyond just stabilizing cellular machinery; it also actively participates in stress-adaptive gene expression networks, which eventually improves productivity and survival in challenging circumstances (Hossain et al., 2021).

GB has been shown to bind to double-stranded DNA *in vitro*, suggesting a potential role in modulating nucleic acid structure and function. Under high-salinity conditions, GB acts as a regulator of gene expression by influencing transcription and DNA replication processes. Transcriptomic analyses using cDNA microarrays have revealed that transgenic plants accumulating GB exhibit altered expression of endogenous genes involved in stress responses, particularly those related to osmolyte biosynthesis and antioxidant defense (Kahraman et al., 2019). In tomato and wheat plants, exogenous application of GB led to significant changes in transcript levels of stress-responsive genes. For instance, GB treatment upregulated the *cat1* gene, which encodes catalase, an essential antioxidant enzyme in tomato, enhancing catalase activity especially under low-temperature stress (Park et al., 2006). Similarly, in wheat, GB influenced the expression of cold-responsive proteins such as *WCOR410* and *WCS120*, which are associated with cellular protection during freezing conditions (Hossain et al., 2022). These findings indicate that GB not only functions as an osmoprotectant but also modulates the transcription of key genes involved in abiotic stress tolerance pathways.

Additionally, gene expression in the flower buds of wild-type and cod-A transgenic tomato was compared using cDNA microarray, wherein the expression of 29 genes was suppressed and the expression of 30 genes was enhanced (Park et al., 2007). Furthermore, Jarin et al. (2024) showed that GB functions similarly to chaperonins *in vivo*. This seems to imply that GB may maintain the translational and transcriptional machinery required for effective gene expression in stressed situations. The ability of GB and its products to activate specific genes in transgenic plants may aid in developing an understanding of GB-enhanced stress tolerance in plants.

### 3.2 GB-mediated protection of photosynthetic machinery

The repair of PSII is inhibited by different abiotic stressors that produce ROS. Heavy metals, low temperatures, mild heat, salinity, and CO_2_ limitation produce ROS by inhibiting CO_2_ fixation and lowering 3-phosphoglyceric acid levels. The amount of NADP^+^ decreases when photosynthetic fixation of CO_2_ is suppressed. Because PSI lacks the primary electron acceptor (NADP^+^), electrons are transported to molecular oxygen more quickly, producing H_2_O_2_ via O_2_^.-^ manufacturing (Chauhan et al., 2023). Consequently, these ROS prevent PSII repair by blocking protein synthesis. Therefore, by preventing PSII repair, ROS increases the degree of photoinhibition. While the repair of the PSII complex entails several procedures that guarantee the removal and replacement of the damaged D1 protein, photodamage to PSII is defined by photochemical damage to a component of the PSII reaction center, namely, the D1 protein (Chauhan et al., 2023).

Furthermore, surplus electrons from PSI are transformed into ROS when photosynthetic fixation of CO_2_ is reduced under abiotic stress. This prevents the translation stage of the pre-D1 protein from being synthesized, which consequently prevents the repair of the photodamaged PSII. GB may maintain the fixation of CO_2_ by shielding CO_2_-fixing enzymes (Rubisco and Rubisco activase) from abiotic stress, which effectively inhibits the generation of ROS. Additionally, GB modulates the genes encoding ROS-scavenging enzymes, which break down ROS and lower their concentrations in cells, thereby lessening the impact of abiotic stress on the photosynthetic apparatus (Didaran et al., 2024). Ohnishi and Murata (2006) documented the interaction between GB synthesis and the effect of salt stress on PSII photoinhibition. While GB, which had been produced *in vivo*, shielded PSII from photoinhibition under these circumstances, salt stress from 220 mM NaCl increased PSII photoinhibition. However, the photodamage to PSII was unaffected by salt stress or GB synthesis. In contrast, GB reversed the inhibitory effect of salt stress, which prevented the effective repair of the photodamaged PSII. According to pulse-chase labeling tests, salt stress decreased both the *de novo* synthesis of the D1 protein and its degradation in photodamaged PSII. However, under salt stress, GB shielded PSII from suppressing D1 protein synthesis and degradation. GB and salt stress had no effect on psbA transcript levels. These findings imply that GB might accelerate the repair of photodamaged PSII by counteracting the inhibitory effects of salt stress.

Furthermore, Yang and Lu (2005) investigated the effects of mild heat stress and GB production in tobacco plants that had been modified to produce GB *in vivo*. Moderate heat stress was observed to block RuBisCO activase, which ultimately restricted the fixation of CO_2_. ROS production was accelerated by these conditions, preventing PSII from being repaired. Rubisco activase is likely protected against moderate heat-induced inhibition by GB. The defense mechanisms of the PSII repair system against mild heat stress and salt stress appear to possess certain characteristics, i.e., the upregulation of protective proteins, enhanced expression and synthesis of the D1 protein, and activation of chloroplast proteases and chaperones that facilitate the efficient degradation and replacement of damaged PSII components. Together, these responses help maintain photosynthetic efficiency by ensuring dynamic recovery of PSII under fluctuating environmental conditions (Nishiyama et al., 2006). Further research is required to thoroughly understand the mechanisms underlying how different types of environmental stressors affect photoinhibition and photosynthesis.

## 4 Role of exogenous GB application in the plant’s abiotic stress tolerance mechanism

**Figure 2:**
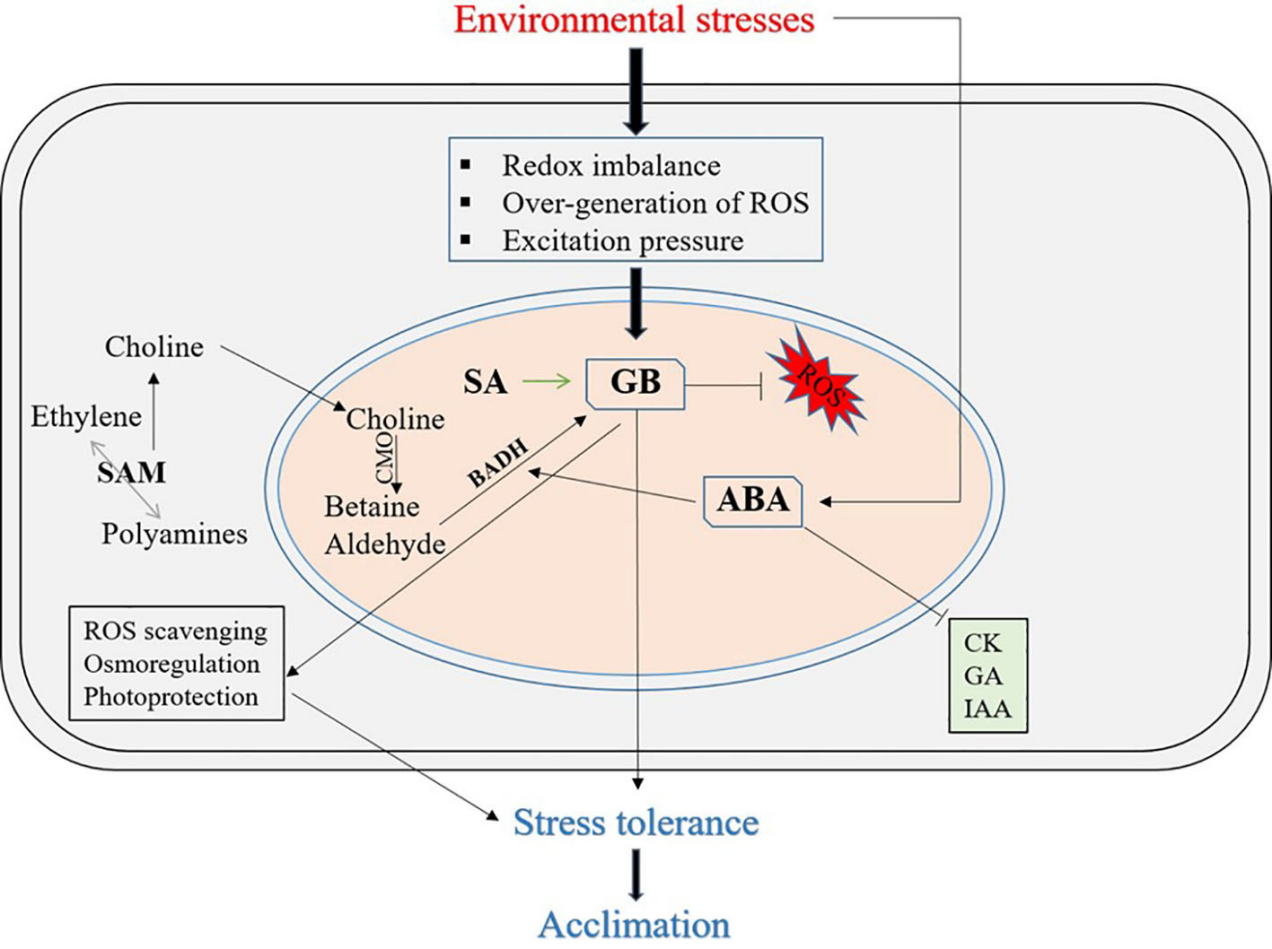
A schematic model illustrating the role of GB in mitigating abiotic stress effects in plants, highlighting its interactions with photosynthesis, plant hormones, and reactive oxygen species (ROS). The figure highlights GB's interactions with key physiological processes under stress conditions, including its influence on plant hormones and reactive oxygen species (ROS). GB helps stabilize cellular structures, protect chloroplasts, and regulate hormonal balance, thereby enhancing the plant’s ability to withstand environmental stressors. Additionally, GB modulates ROS scavenging mechanisms, preventing oxidative damage and maintaining osmoregulation. ROS, Reactive Oxygen Species; SAM, S-adenosylmethionine; SA, Salicylic Acid; CMO, Choline Monooxygenase; CK, Cytokinins; GA, Gibberellins; IAA, Indole-3-acetic acid; ABA, Abscisic Acid; BADH, Betaine Aldehyde Dehydrogenase.

Numerous environmental stressors typically affect the growth, development, yield, and productivity of different plants. Abiotic stress has become a serious global issue that significantly limits the yield of food and economic crops (Liang et al., 2024). Plants have evolved a number of natural stress-protective mechanisms to combat abiotic stress (**Figure 2**). However, plants cannot withstand or adapt to higher levels of abiotic stressors or many abiotic stresses (Ghorbani et al., 2023). Therefore, depending on the crops and their diverse species, farmers use a variety of exogenous treatments with different agrochemicals, pesticides, PGRs, growth stimulators, osmolytes, or osmoprotectants, among others, to ensure that plants survive harsh conditions. The crucial role of the accumulation of osmoprotectants, such as GB and proline, in osmotic adjustments and considerable improvements in plant stress physiology under stress- and tissue-specific regulation in response to multiple abiotic stresses has now been fully documented (Dutta et al., 2019).

It has been found that GB foliar application topically to onion plants increases their resistance to salt stress by boosting their antioxidant defense systems, which are essential for reducing oxidative damage and scavenging reactive oxygen species (ROS). Furthermore, GB strengthens the plant’s defensive mechanisms by encouraging the accumulation of non-enzymatic antioxidants such as glutathione, ascorbic acid, and phenolic substances. Lower levels of malondialdehyde (MDA) indicate a reduction in lipid peroxidation, which GB helps to maintain membrane integrity and protects against cellular damage under salt stress. Additionally, it prevents excessive ion toxicity by controlling the balance of potassium (K^+^) and sodium (Na^+^), which helps maintain ion homeostasis. By these means, GB helps onion plants better tolerate saline conditions by reducing oxidative stress and promoting general plant development and physiological performance (Rady et al., 2018). Exogenous GB supplementation has been shown to reduce salt stress in *Phaseolus vulgaris* (common bean) by preserving a high K^+^/Na+ ratio, limiting the uptake of Na^+^ ions, and promoting antioxidant defense mechanisms to reduce salinity-induced toxicity. However, by improving K^+^ uptake and maintaining a higher K^+^/Na^+^ ratio, GB treatments simultaneously produced salt stress tolerance, which in turn caused the common bean plants to experience osmoregulation (Sofy et al., 2020). Additionally, it was found that GB-induced salt stress protection in common beans is more closely linked to ion uptake regulation than to the capacity of the antioxidant defense system (**Table 1**). It is stated that GB stabilizes plasma membrane integrity and transporter proteins, thereby reducing nonspecific ion influx and leakage (Chen and Murata, 2008; Chen and Murata, 2011). Also, GB modulates the activity and expression of specific ion transporters and channels, such as H^+^-ATPases and metal transporter families (ZIP, NRAMP), enhancing selective uptake and sequestration of essential ions while limiting toxic metal accumulation (Hasanuzzaman et al., 2020). Additionally, GB enhances antioxidant defense systems, indirectly supporting ion homeostasis by protecting transporters from oxidative damage (Ali et al., 2020). These combined effects enable plants to maintain nutrient uptake and reduce heavy metal toxicity under stress conditions.

Furthermore, GB has been shown to control several cellular metabolism-related functions in addition to acting as an osmolyte (Figueroa-Soto and Valenzuela-Soto, 2018). GB plays a major role in protein synthesis and is actively involved in the control of homocysteine/methionine pathway enzymes, metabolism of carbohydrates (glucose, sucrose, and fructose), glycogen, lipogenesis, and lipid oxidation. Based on previous research on the underlying mechanisms of GB actions, GB may alter the phosphorylation status of certain kinases or cause changes in the methylation status of target gene promoters to alter the activities of enzymes. By enhancing growth, development, photosynthesis, antioxidant enzyme activity, nutrient or mineral uptake, regulating antioxidant levels, and lowering the uptake of excessive heavy metals, GB has been proposed to enable plants to withstand heavy metal stress, thereby limiting oxidative stress (Ali et al., 2020).

In addition, Huang et al. (2020) determined that GB increases PSII’s oxygen-evolving activity of PSII, in addition to stabilizing its structure. The oxygen-evolving complex (OEC), which is essential for effective water splitting and oxygen synthesis, is kept intact in part by GB. GB shields PSII from oxidative degradation and stops photoinhibition by stabilizing important PSII proteins like D1 and D2. Furthermore, GB ensures the best possible electron transfer efficiency by assisting in the maintenance of the correct configuration of PSII-associated cofactors, such as manganese (Mn) clusters. In the end, this structural stabilization improves energy generation and photosynthetic efficiency, enabling plants to maintain improved growth and stress tolerance. Under drought stress conditions, GB treatment remarkably decreased the levels of hydrogen peroxide, superoxide ions, and malonaldehyde in maize. Moreover, it has been shown that GB treatment increases maize resistance to salinity stress by controlling physiological characteristics, ionic homeostasis, and antioxidant defense systems (Dustgeer et al., 2021). A recent study by Zulfiqar et al. (2022) emphasized the key role of GB in providing plant thermotolerance and claimed that exogenous GB treatments, such as seed priming or plant priming, increase endogenous levels of GB, which helps plants to tolerate heat stress. Likewise, it has been stated that foliar application of GB greatly improves the morphology, growth, chlorophyll levels, relative water content, and gas-exchange characteristics of maize under conditions of water deficit, with the exception of intercellular CO_2_ concentration, osmolytes, ROS accumulation, antioxidant enzyme activities, and yield (Shemi et al., 2021). Wang et al. (2024) recently demonstrated that GB synthesis by genetic engineering might increase the tolerance of tobacco photosynthetic apparatus against drought stress. This study compared the ability of transgenic tobacco to withstand drought stress by accumulating GB in wild-type tobacco plants. By measuring photosynthetic gas exchange parameters, thylakoid membrane protein function, chloroplast structure, thylakoid membrane/lamellae structure or stability, and chlorophyll fluorescence, it was discovered that transgenic tobacco improved the photosynthetic apparatus’s resistance to drought. In addition, through greater oxidation of the xanthophyll cycle, genetic engineering of GB synthesis could improve the unsaturated fatty acid index, increase digalactosyl diacylglycerol and phosphatidylglycerol levels, and reduce the photoinhibition of PSII in tobacco exposed to drought stress.

## 5 GB cross-talk with phytohormone and signaling molecules

Phytohormones are the primary signaling molecules in plants and play a crucial role in controlling abiotic stress responses in all plant species. In essence, stress hormones, such as ABA, ethylene, salicylic acid (SA), and other conventional plant growth regulators (PGRs), play a role in controlling plant signaling (Basit et al., 2021; Aerts et al., 2021). Other molecules, such as charged PAs, the gasotransmitter nitric oxide (NO), and even plant nutrients, balance the cellular environment when the body reacts to unfavorable conditions (Wu et al., 2024). During abiotic stress, glycine betaine stabilizes the cellular osmoticum and detoxifies ROS molecules by inducing antioxidant machinery. This process requires complex interactions between signaling-related molecular components (**Table 3**).

**Table 3:** Crosstalk between glycine betaine and phytohormones/signaling molecules in regulating plant stress responses.

Phytohormone and Signaling Molecules	Crosstalk with GB	GB-Mediated Physiological Responses under Abiotic Stresses	References
Abscisic Acid (ABA)	GB enhances ABA biosynthesis and signaling under drought and salt stress. GB also stabilizes ABA receptors.	Improved stomatal regulation, drought, and salt tolerance	(Chen and Murata, 2011; Zhou JinXin et al., 2008)
Salicylic Acid (SA)	GB modulates SA-dependent defense pathways; synergistic action in oxidative stress management.	Enhanced systemic acquired resistance (SAR) and antioxidative enzyme activity	(Aerts et al., 2021)
Melatonin	GB and melatonin synergistically enhance antioxidant defense and photosynthetic efficiency.	Improved tolerance to abiotic stresses like salinity and heavy metals	(Lv et al., 2021)
Jasmonic Acid (JA)	GB prime JA biosynthesis/signaling under stress. Crosstalk results in enhanced secondary metabolite production.	Improved resistance to biotic stress and ROS scavenging	(Farooq et al., 2024)
Hydrogen Sulfide (H₂S)	Limited studies; potential interaction via redox balance and antioxidant signaling.	Under exploration for salt and drought stress responses	(Li et al., 2021)
Nitric Oxide (NO)	GB modulates NO levels; NO acts as a secondary messenger for GB-induced stress responses.	Regulation of antioxidant systems and stomatal behavior	(Zhou et al., 2021)
Brassinosteroids (BRs)	BRs and GB act synergistically to enhance antioxidative defense and stress tolerance.	Membrane stabilization and improved enzyme activity	(Kang et al., 2024; Nolan et al., 2017)
Ethylene (ET)	GB reduces ET overproduction under stress, modulating senescence and improving photosynthesis.	Delayed leaf senescence, enhanced stress tolerance	(Bansal and Wani, 2021; Song et al., 2014)

### 5.1 Phytohormones

#### 5.1.1 Ethylene

The gaseous hormone ethylene is necessary for plant growth and development (Khan et al., 2024). Additionally, by encouraging stem elongation, leaf area expansion, stem radial growth, and leaf thickness, ethylene increases the resistance to extreme forms of freezing that are relatively severe (Kurepin et al., 2017). As choline, a common mediator molecule, is involved, ethylene and GB production are closely related. A methyl group is transferred from S-adenosyl methionine (SAM) to choline, which facilitates ethylene production. Therefore, the production of ethylene may occur at the expense of GB synthesis or alternatively. To ensure development and survival, plants under extreme abiotic stress conditions produce endogenous GB (Wang et al., 2024). This may occur at the expense of ethylene production because choline is channeled into GB synthesis. Tobacco and tomatoes, which are high ethylene-accumulating species, did not accumulate large amounts of GB under cold or other stresses. Additionally, in response to abiotic stresses, species with minimal ethylene accumulation showed higher GB production (Kurepin et al., 2017).

#### 5.1.2 Abscisic acid

Abscisic acid (ABA) is considered a universal stress phytohormone that regulates a wide range of physiological processes under abiotic stressors. In response to stress, plant tissues assemble relatively high levels of ABA (Roychoudhury and Banerjee, 2017). In addition to preventing unintended desiccation and water loss, this enables quick stomatal closure. Exogenous ABA treatment promotes GB accumulation in barley plants under drought, salt, and low-temperature stresses (Jagendorf and Takabe, 2001; Holmström et al., 2000). An intriguing study demonstrated that the exogenous application of either ABA or GB improved the ability of Arabidopsis to withstand freezing temperatures. Nevertheless, GB production was boosted by ABA-only treatment, suggesting that ABA may function upstream of the GB biosynthesis pathway. An investigation regarding the temporal regulation between ABA and GB determined that drought stress initially augmented the endogenous ABA content in the cells of *Zea mays* seedlings, followed by BADH transcription and GB content (Zhang et al., 2012). Reduced photosynthetic activity may result from the efficient induction of stomatal closure caused by ABA accumulation under abiotic stress. Nonetheless, the functions of GB in enhancing photosynthetic efficiency under abiotic stress have been addressed earlier. The roles of ABA and GB might seem incompatible because ABA positively regulates GB production. However, exogenous ABA application decreased stomatal conductance without decreasing *Tradescantia virginiana*’s photosynthetic ability, according to the investigation of Franks and Farquhar Franks and Farquhar (2001). This inference most probably emphasizes the correlated functions of ABA and GB in response to abiotic stresses.

#### 5.1.3 Salicylic Acid

Abiotic stress tolerance in plants is also linked to endogenous salicylic acid (SA) accumulation. Salicylic acid promotes the synthesis of both enzymatic and non-enzymatic antioxidants, which effectively scavenge ROS and provide a defense against oxidative stress (Salinas et al., 2025). Additionally, exogenous SA supplementation has helped to develop tolerance to drought, salt, heavy metal toxicity, and temperature extremes (Paul et al., 2024). In contrast to the amount of grain production in the stressed plants treated separately with SA or GB, Aldesuquy et al. (2012) demonstrated that the co-application of SA and GB further increased the yield in drought-stressed wheat plants.

#### 5.1.4 Other Traditional Plant Growth Regulators

Auxins, gibberellic acids (GAs), and cytokinins (CKs) are traditional plant growth regulators (PGRs). They are involved in growth, development, stem elongation, polar solute transport, phototropism and gravitropism, cell division, and initiation of reproduction (Singh et al., 2024). ABA and GA are known to have antagonistic relationships. GA production is suppressed during abiotic stress when endogenous ABA levels increase (Kurepin et al., 2017). As GB synthesis is triggered by ABA accumulation, it may be concluded that GB and GA have a negative relationship. Bao et al. (2011) noticed that transgenic *Lolium perenne* plants that overexpressed *CMO* and *BADH* genes from spinach chloroplasts had reduced GA1 levels, which is consistent with this hypothesis. Additionally, there was a negative correlation between cytokinin levels and GB accumulation. Kathuria et al. (2009) found that transgenic rice plants overexpressing choline oxidase (*coda*) showed higher production of CK *dehydrogenase 1* (*CKX1*), an enzyme that breaks down CK. Plants had low CK concentrations and significant GB accumulation. GB synthesis is also negatively regulated by auxin. In *Kochia scoparia* plants subjected to salt stress, exogenous administration of the synthetic auxin dicamba decreased *CMO* expression and, consequently, GB accumulation (Kern and Dyer, 2004).

### 5.2 Polyamines

Glycine betaine and polyamine crosstalk as compatible solutes to control the cellular osmoticum during abiotic stressors (Paul et al., 2017). GB is associated with polyamines, such as putrescine, spermidine, and spermine, because they perform their corresponding functions. Prior to salt treatment, seedlings primed with spermidine and spermine exhibited higher levels of *BADH1* expression in the shoots of the salt-tolerant rice cultivar Nonabokra than in the salt-sensitive rice cultivar IR-64. This was in contrast to seedlings that were not primed under stress. Conversely, priming with spermidine and spermine promoted the accumulation of *BADH1* transcripts in Nonabokra roots and decreased gene expression in IR-64 roots subjected to 75 mM NaCl (Banerjee and Roychoudhury, 2018).

In the roots and shoots of Gobindobhog, another salt-sensitive, aromatic rice cultivar, polyamine priming of salt-stressed seedlings increased *BADH1* expression (Paul and Roychoudhury, 2017). These findings unequivocally demonstrate how PAs and the mechanism involved in GB biosynthesis interact when rice is subjected to salt stress. Under salt stress, the amount of GB in Arabidopsis increases due to the overexpression of *aldehyde dehydrogenase 10A8* (*ALDH10A8*) and *ALDH10A9* (encoding functional BADH). Additionally, transgenic plants showed a decrease in putrescine and spermidine content during salinization, as well as an increase in free PAs such as agmatine, cadaverine, and tyramine (Rippa et al., 2012). The ability of BADHs to oxidize 3-aminopropanal, an intermediate molecule in PA catabolism, was demonstrated by Rippa et al (Bansal and Wani, 2021). 3-Aminopropanal is produced when polyamine oxidase (PAO) oxidizes spermine, and ALDH10A8 and ALDH10A9 are used as substrates (Missihoun et al., 2015). This indicated the direct involvement of BADH isoforms in spermine degradation. Choudhary et al. (2012) reported that exogenous supplementation with spermidine and epibrassinolide (an active brassinosteroid) markedly elevated the titers of cellular antioxidants, such as GB, Pro, AsA, GSH, and total phenolics, in *Raphanus sativus* seedlings subjected to Cr stress. This led to the effective scavenging of excess ROS and the acquisition of stress tolerance. This demonstrates that PAs, GB, and brassinosteroids are regulated in three steps to induce stress-induced responses. When *Stevia rebaudiana*, a perennial medicinal herb, was supplemented with synthetic PA (diamino hydroxyl ethyl phospho mineral and diaminohexanoic mineral amino ethanoic acid), the endogenous GB and Pro levels amplified in response to cold stress (Moradi Peynevandi et al., 2018).

### 5.3 Gasotransmitters

Nitric oxide (NO) and hydrogen sulfide (H_2_S) are gaseous chemicals involved in various plant-signaling systems. Additionally, these gasotransmitters help plants to become more resilient to multiple abiotic stressors (Basit et al., 2023). They are essential, concentration-dependent redox-related signaling molecules (Basit et al., 2024a). Cellular generation of H_2_S was elevated in *Spinacia oleracea*seedlings when sodium hydrosulfide (NaHS) was applied exogenously. Following NaHS treatment, there was an increase in the transcription of *BADH* and *CMO* genes. In treated plants, the H_2_S-induced buildup of GB programmable the antioxidant machinery and enhances drought resistance (Chen et al., 2016). Unknown signaling connections between H_2_S and GB that have not been thoroughly studied have been suggested by these studies. Zanganeh et al. (2018) recently demonstrated that seed priming with SA and NaHS caused GB and NO accumulation in *Z. mays* seedlings grown under Pb stress. Additionally, treated plants showed decreased expression of *1-aminocyclopropane-1-carboxylate synthase 6* (*ACS6*), a gene involved in ethylene biosynthesis. Choline channelization toward GB synthesis rather than ethylene generation was the cause of this phenomenon. Arginine metabolism is most likely directed toward NO formation rather than PA synthesis, as evidenced by the decrease in the PA biosynthesis gene, *SAM decarboxylase* (*SAMDC*), and the generation of NO during Pb stress (Zanganeh et al., 2018). Therefore, the combined effects of H_2_S, NO, and GB on Pb tolerance in maize plants were investigated. GB and NO have a positive regulatory relationship in maize seedlings under oxidative stress (Phillips et al., 2018). Nitric oxide synthase (NOS) inhibitor-treated seedlings showed decreased *BADH* expression and increased GB. As a result of decreased GB production, the activity of antioxidant enzymes is also inhibited. In seedlings treated with NOS inhibitors, there was an increase in H_2_O_2_ accumulation due to a decrease in CAT expression (Phillips et al., 2018).

## 6 Plant engineering to improve GB biosynthesis

Abiotic stresses can reduce plant growth, yield loss, and even plant death, severely affecting agricultural productivity. Factors, such as prolonged drought, high salinity, and extreme temperatures, disturb plant metabolism and cause cellular damage, including oxidative stress, dehydration, and ionic imbalances. Recent biotechnological advancements, particularly in the regulation of molecular targets and the development of transgenic plants, have facilitated a deeper understanding of GB-mediated tolerance to abiotic stress. However, the molecular biology of GB production has not yet been thoroughly explored. Recent studies have confirmed the molecular mechanisms underlying GB accumulation in response to stress. Proteomic studies have identified BADH as a marker for salt stress in *T. monococcum* seedlings (Lv et al., 2016). The proteome of the seedlings subjected to salt stress was examined using two-dimensional gel electrophoresis (2-DE) in conjunction with MALDI-TOF-TOF mass spectrometry. This analysis led to the identification of proteins associated with regulatory functions, stress protection, protein dynamics, and metabolic pathways. Subsequent investigations have confirmed that BADH, leucine aminopeptidase 2, Cu/Zn superoxide dismutase, and 2-Cys peroxiredoxin BAS1 serve as molecular markers indicative of the response to salt stress (Hasanuzzaman et al., 2019). The significance of BADH in abiotic stress tolerance was demonstrated through a knockdown experiment employing RNA interference (RNAi). In rice, *BADH1*-*RNAi* repression lines showed diminished yield and reduced tolerance to salt, drought, and cold conditions. This phenomenon has been attributed to increased levels of MDA and H_2_O_2_ accumulation in plant tissues (Tang et al., 2014). Notably, the repression lines did not exhibit alterations in the endogenous GB content. Therefore, the observed decline in stress tolerance may be linked to decreased dehydrogenation of aldehydes that accumulate in various metabolic pathways.

Major cereal crops, including barley, wheat, and maize, exhibit limited capacity to accumulate GB under normal conditions. Consequently, transgenic strategies aimed at overexpressing genes involved in GB biosynthesis have been implemented to improve plant resilience to abiotic stress. Rice, a key staple food crop, possesses one *CMO* and two *BADH* homologs (Baicharoen et al., 2018; Ghosh and Roychoudhury, 2018; Hernandez-Leon and Valenzuela-Soto, 2023). However, the deletion of the translational initiation codon, loss of essential functional domains, and emergence of premature stop codons due to frameshift mutations collectively lead to the production of non-functional *BADH2* homologs. This gene plays a crucial role in the synthesis of the aromatic compound 2-acetyl-1-pyrroline found in aromatic rice varieties (Baicharoen et al., 2018). In contrast, the functional *BADH1* homolog is involved in the production of GB under osmotic stress (Ghosh and Roychoudhury, 2018). Genetic engineering of GB biosynthesis in GB non- or low-accumulators has been tested in diverse plant species, including Arabidopsis, tobacco, Brassica, tomato, maize, rice, potato, and wheat. This results in an increased tolerance to multiple stresses (Sharma et al., 2024). The association between ion channel regulation and GB has been documented by Ahire et al. (2018). This study demonstrated that overexpression of *vacuolar proton pyrophosphatase* (*VPPase*) derived from *Sorghum bicolor* in *Bacopa monnieri* resulted in increased salt tolerance, which was attributed to the enhanced production of GB. Furthermore, transgenic plant shoots showed reduced levels of malondialdehyde (MDA) and exhibited diminished membrane damage, which can be ascribed to the GB-mediated activation of the antioxidant system and the efficient scavenging of harmful reactive oxygen species (ROS) (Ahire et al., 2018).

Overexpression of genes responsible for stress-related proteins has been shown to stimulate the synthesis of GB in transgenic plant tissues (Zulfiqar et al., 2022). For instance, the introduction of the tobacco *osmotin* gene into chilli pepper plants led to improved salt tolerance, which was attributed to the increased production of GB and proline. These transgenic plants demonstrated elevated activities of superoxide dismutase (SOD), glutathione reductase (GR), and ascorbate peroxidase (APX), along with a higher relative water content (RWC) under stress conditions when compared to control plants (Subramanyam et al., 2011). Additionally, the G-protein RabAc4, which plays a role in membrane trafficking, has been identified to work in conjunction with GB to enhance chilling tolerance in tomato seedlings (Subramanyam et al., 2011). Summary of transgenic strategies aimed at boosting GB accumulation and enhancing tolerance to abiotic stress.

## 7 Conclusion and future perspectives

Glycine betaine, the principal organic osmolyte, plays multiple roles in plant growth and development. Many higher plants naturally synthesize GB as a protective measure in response to various environmental stresses. In addition to its function as an osmoprotectant, GB plays a crucial role in stabilizing cytosolic pH, proteins, enzymes, and membranes while also scavenging harmful ROS and preserving the redox balance within cells, thereby protecting subcellular structures in plants under stress. Furthermore, GB helps maintain ionic equilibrium in plants under different stress conditions. Recent research has extensively demonstrated that both endogenous and exogenous GB enhance plant tolerance to various abiotic stress events. Recent research suggests that differentially expressed endogenous genes play a crucial role in the stress tolerance of higher plants mediated by GB. This process involves scavenging of ROS, management of oxidative stress, modulation of gene expression, and enhanced accumulation of GB under abiotic stress conditions. Owing to the diverse functions of the genes involved in the GB biosynthesis pathway, efforts are underway to create transgenic plants that can efficiently accumulate GB, thus exhibiting improved resilience to various abiotic stresses, including secondary oxidative bursts. Despite ongoing research on GB, its signaling mechanisms remain poorly understood and require further exploration.
